# Listening to Women's Voices: A Patient and Public Involvement Exercise Exploring Vulval Reconstructive Surgery for UK Women With Female Genital Mutilation (FGM)

**DOI:** 10.1111/hex.70275

**Published:** 2025-05-12

**Authors:** Juliet Albert, Janet Fyle, Njomeza Kartallozi, Christie Coho, Naana Otoo‐Oyortey, Hekate Papadaki, Catrin Evans, Dalia Saidan, Sohier Elneil, Natasha Anderson‐Foster, Jasmine Abdulcadir, Huda Mohamed, Aurora Almadori

**Affiliations:** ^1^ Imperial College Division of Womens, Children and Clinical Support, Imperial College Healthcare NHS Trust (ICHNT) and Imperial College London London UK; ^2^ Royal College of Midwives London UK; ^3^ Dahlia Project, Manor Gardens London UK; ^4^ Independent Psychological Therapist London UK; ^5^ FORWARD London UK; ^6^ School of Health Sciences, Centre for Evidence Based Healthcare University of Nottingham Nottingham UK; ^7^ Croydon University Hospital Urogynaecology and Pelvic Floor Reconstruction Unit London UK; ^8^ Institute for Women's Health University College London London UK; ^9^ Psychosexual Therapist in Private Practice Birmingham UK; ^10^ Hôpitaux Universitaires de Genève (HUG) clinic Geneva Switzerland; ^11^ Whittington Hospital London UK; ^12^ Division of Surgery and Interventional Science University College London London UK

**Keywords:** co‐production, equality, diversity and inclusion, female genital mutilation, health inequalities, patient and public involvement and engagement, reconstruction surgery, underserved communities

## Abstract

**Introduction:**

This article presents patient and public involvement and engagement (PPIE) work undertaken to explore FGM survivors' and stakeholders' views on reconstructive surgery, potential service models, care pathways, barriers to access and other support needs. The aim was to set research priorities, identify key themes and help inform subsequent research in the field.

**Methods:**

A national research collective was established comprising over 20 stakeholders, including FGM survivors/women with lived experience, healthcare professionals, academics and advocacy groups. The group undertook two discussion workshops with FGM survivors (*n* = 11 participants), two national stakeholder events (*n* = 142 attendees) and significant advocacy and partnership‐building activities.

**Results:**

Key insights were that FGM survivors would value reconstructive surgery to address body image concerns, genital pain and sexual difficulties. Potential barriers to surgery included stigma, safeguarding concerns, lack of awareness and fear. Significant gaps were identified around women's knowledge of clitoral anatomy, FGM types and specialist services. Survivors and stakeholders emphasised the need to complement surgical reconstruction with a comprehensive care pathway including trauma counselling and psychosexual therapy.

**Conclusion:**

This study highlights the importance of a survivor‐led approach to FGM service development, as often the voices of FGM survivors are not included. The exercise demonstrated that, with the right approach, it is possible to engage ‘minoritised communities/individuals from the global majority’ and communities dispayed considerable willingness to participate in this sensitive research field. It also emphasises an urgent need for accessible, high‐quality FGM care informed by the voices of those affected, to improve outcomes and support for FGM survivors in the United Kingdom.

**Patient or Public Contribution:**

Women with lived experience of FGM and women from FGM‐affected communities, as well as other national stakeholders (including Non Government Organisation's and charities working with FGM survivors, academics, artists and campaigners were involved in the design and conduct of this study, analysis and interpretation of the data and preparation of the manuscript.

## 1 Background

### 1.1 FGM in the United Kingdom

Female genital mutilation (FGM) involves the partial or total removal of the external genitalia for non‐medical reasons [1, 2]. It offers no health benefits and violates several human rights principles [3]. Long‐term physical consequences include sexual health problems (such as decreased desire and pleasure, painful sexual intercourse, decreased lubrication, reduced frequency or absence of orgasm), excessive scar formation, childbirth complications and psychological problems (including post‐traumatic stress disorder, anxiety, depression and body dysmorphia) [2, 4]. Additionally, women are often left with genital scars leading to symptoms such as pain, itching and increased risk of tearing during vaginal childbirth [3].

In the United Kingdom, FGM is a major public health issue. Now somewhat dated, the most recent prevalence study (produced in 2015) estimates 137,000 FGM survivors in England and Wales [5]. Between 2015 and 2023, NHS England records show 33,620 women and girls with FGM made 87,575 attendances to hospital trusts, mental health trusts or GP surgeries [6]. FGM‐related health issues cost the NHS around £100 million annually, with 65% for psychological and psychosexual problems and 33% for long‐term physical consequences [7]. However, there are acknowledged limitations in the comprehensiveness and accuracy of these data [8].

Currently, there are 28 NHS FGM specialist clinics in the United Kingdom [9]. Activities include FGM diagnosis, safeguarding assessments, trauma counselling, health advocacy and deinfibulation (opening surgery for women with Type 3) [10]. Some clinics also provide surgeries for voiding dysfunction, scarring and cysts. However, psychosexual counselling is rarely offered, and reconstruction surgery is not available. Recent evidence suggests that women's experiences of care within specialist services can be variable and that a ‘postcode lottery’ exists [11]. Moreover, within the wider political context, recent evidence from UK maternity services has demonstrated that women from the global majority are doubly marginalised within the NHS by gender and skin colour [12].

#### 1.1.1 Reconstructive Surgery

Vulval reconstruction related to FGM includes techniques to address different anatomical units of the vulva, usually removing scar tissue to reveal remaining underlying clitoral tissue and/or rebuilding the clitoral glans and/or hood, and/or inner labia, with clitoral reconstruction being the most common procedure. Introduced in France in 1998 [13], it is available in several European countries (including Spain, France, Germany, Belgium, Switzerland, Sweden and the Netherlands), Africa (Kenya, Burkina Faso and Egypt), and the United States.

In 2015, the Royal College of Obstetricians and Gynaecologists (RCOG) produced guidelines stating, ‘*clitoral reconstruction should not be performed because current evidence suggests unacceptable complication rates without conclusive evidence of benefit*’ [10]. They recently updated the guidance, in 2024, acknowledging the need for well‐designed randomised clinical trials (RCTs). In 2016, the World Health Organization (WHO) ‘Guidelines on the Management of Health Complications from FGM’ similarly declined to recommend reconstruction surgery, yet stated ‘*evidence indicates that reconstructive clitoral surgery can improve chronic clitoral pain as well as dyspareunia symptoms*’ [2]. Since these publications, FGM reconstruction research has moved on significantly, and new access and funding for reconstruction surgery in the Netherlands provide an example of how advocacy and collaboration can result in policy change [14]. Recent systematic reviews show the evidence base is growing, collectively suggesting positive outcomes and minor complication rates [15, 16, 17, 18, 19].

Anecdotal evidence and limited research suggests UK FGM survivors are increasingly seeking reconstruction, often travelling abroad for the procedure if they can afford it [20, 21, 22]. A 2024 BBC article reported news of a UK survivor who underwent surgery abroad, demonstrating that this issue has now reached mainstream media [23]. Travelling overseas for surgery represents both an emotional and financial burden for women and leads to potential additional NHS costs if post‐surgical complications arise [24]. It is unknown how many UK survivors have had, or may seek, reconstruction, but French surgeon Dr. Pierre Foldes estimates over 60 UK women have undergone clitoral reconstruction in France, with another 20 treated by Dr. Cornelia Strunz in Germany since 2013 (via email correspondance).

#### 1.1.2 FGM Stakeholders—ACERS‐UK

The authors of this paper are members of ‘ACERS‐UK,’ a female‐led voluntary collective advocating for a national Centre of Excellence offering Access to Clitoral Reconstruction and Emotional Support for FGM survivors within a research framework. Co‐founded by (J.A.) and (S.E.) in 2021, the collective includes FGM survivors, healthcare professionals (specialist midwives, urologists, uro‐gynaecologists, trauma and psychosexual therapists, health advocates, a gynaecologist, plastic surgeon and a GP), FGM policy advisor from the Royal College of Midwives, President of the Faculty of Sexual and Reproductive Health; academics; activist/campaigners and charity representatives from Foundation for Women's Research and Development (FORWARD UK), Dahlia Project (Manor Gardens) and Sister Circle. A webpage is hosted by the RCM FGM network (https://fgmnetwork.org.uk/fgm-reconstruction-surgery/). Furthermore, the project has strong partnerships with other anti‐FGM organisations, including Midaye, Iranian & Kurdish Women's Rights (IKWRO), INTEGRATE‐UK, NESTAC, Sundial Centre for Education on Harmful Practices, Barnardo's National FGM Centre and the Vavengers.

The collective meets remotely every month outside of working time to discuss ideas and update the work plan. Recognising the need for a strong evidence base to conduct research and achieve policy changes, the group is working to develop a co‐designed research programme to assess aspects of genital reconstructive surgery for FGM survivors. This paper outlines the first step of this programme, involving extensive patient and public involvement and engagement (PPIE) to gather stakeholders' and survivors' views.

#### 1.1.3 PPIE

PPIE is an essential component of clinical research. It is argued PPIE input improves the relevance and quality of research (and subsequent service development), by ensuring that these focus on issues of importance to patients [25], highlighting that clinical developments should be carried out ‘with’ or ‘by’ members of the public, rather than ‘to’, ‘about’ or ‘for’ them [26, 27]. Previous initiatives demonstrate the importance of involving FGM survivors in setting the research agenda [28, 29] and the role PPIE can play in addressing disparities and inequalities in women's healthcare [30, 31, 32, 33, 34]. There is a recognised need to develop different ways of engaging and working with ‘racially minoritised’ or otherwise vulnerable communities. Our project provides an example of good practice in PPIE conduct, adopting the principle that ‘*individuals have a right to make decisions about treatments and manage their own health*’ [21].

#### 1.1.4 Terminology

In this paper, women with lived experience of FGM are referred to as ‘survivors’ rather than victims, in line with ‘End FGM’ European network recommendations [35]. We use the definition ‘mutilation’ in line with the United Nations, as it ‘*embraces a human rights perspective on the issue and is used in a number of UN and intergovernmental documents’* [36]. However, we acknowledge the term was coined by Western feminist activists and carries specific cultural and historical connotations that may have different meanings across cultures. We use ‘racially minoritised communities’ to reflect the fact that the United Kingdom is a country where the global majority [37] still experience serious health inequalities. The term ‘minoritised’ highlights the ‘*active processes involved in differential allocations of power, resources and ultimately health’* [38]. Use of the term ‘global majority’ shifts the focus from a deficit perspective to one of representation and therefore increases accuracy and inclusivity. However, our context‐specific approach ensures that we do not misrepresent the reality of the experience that is the norm within the United Kingdom and other countries of the Global North, where the global majority are still labelled and treated as ‘other’/'nonwhite’/'minority’. In some places, we use the term ‘vulnerable communities’, which may be perceived as implying a lack of agency. However, we use the term ‘vulnerable’ to describe the structural disadvantages and health inequalities that many FGM survivors face. We recognise the strength and resilience of these communities, and the term is intended to highlight the need for additional support and advocacy to address these inequities.

## 2 Aims

Our overall aim was to better understand the motives and attitudes of FGM survivors towards surgical reconstruction and to inform future research on FGM reconstructive surgery. Additionally, we sought to develop patient‐relevant research questions regarding the design of potential future clinical trials.

The PPIE activities had three specific objectives:

i. Understand the motivations or barriers to women seeking reconstructive surgery.
ii. Explore research and service needs from a range of stakeholders' perspectives.
iii. Involve survivors in the co‐design of future services.

## 3 Methods/Process

This study was developed in adherence with the Guidance for Reporting Involvement of Patients and the Public (GRIPP2) (Appendix 1) and followed the EQUATOR network high‐standard reporting guidelines [32, 33].

We undertook a series of London‐based events engaging FGM survivors, members of FGM‐affected communities, charity/Non Government Organisation (NGO) community engagement workers, activists/campaigners, academics and healthcare professionals from across the United Kingdom. These events were co‐produced with input from the entire ACERS team, drawing on the multidisciplinary expertise in planning, hosting and facilitating such events. PPIE was structured in sequential components: (i) face‐to‐face discussion workshops with six FGM survivors in November 2023; (ii) national stakeholder event in February 2024 (*n* = 59); (iii) second face‐to‐face discussion workshop with five survivors in May 2024 and (iv) follow‐up stakeholder event in September 2024 (*n* = 83).

After obtaining verbal consent, all conversations were audio‐recorded, and transcripts were anonymised. Scripts were scrutinised and analysed by an academic plastic surgeon (A.A.) utilising a descriptive content analysis approach [39]. At all times, data was stored and shared in a secure way. This approach is well‐suited to the early stages of PPIE work as it provides a flexible and accessible method for analysis that can be inductive or deductive. The main approach in this project was a deductive analysis, summarising the key insights into categories related to the main lines of questioning. This helped the team to clearly identify the key PPI insights for further project development.

### 3.1 Face‐to‐Face Discussion Workshops

Two small discussion workshops were undertaken to hear women's voices. The format ensured this intimate subject could be discussed by women among their peers, recognising the culturally sensitive nature of the topic and ensuring confidentiality. The workshops were organised with the Dahlia Project [40], run by Manor Gardens Welfare Trust, which provides psychotherapy, advocacy, education and post‐therapy empowerment sessions within a dynamic community development model. Their lead psychological therapist (N.K.) distributed a leaflet inviting FGM survivors already accessing therapy groups at the Centre to join the first workshop (Appendix 2). FGM survivors participated voluntarily. Lunch, refreshments and a creche were provided, along with £50 thank you vouchers. Questions were developed in consultation with a social worker, and feedback was incorporated from ACERS members. Women completed a form with their demographic information and were reassured that all information recorded was anonymised. At the workshop, FGM survivors and healthcare professionals—FGM specialist midwife (J.A.) and FGM psychological therapist experts (N.K. and C.C.)—sat together around a table. Women were asked eight open‐ended questions and given the chance to comment freely (Table 1A). The second discussion workshop was repeated 4 months later with different survivors (and C.C. replaced by D.S.) to validate and further explore the previous responses.

**Table 1:** Patient and public involvement and engagement (PPIE) questions.

A. Focus Groups—Discussion Questions	
1.	Who do women talk to about these issues (FGM, sexual pleasure, etc.)?
2.	Why do you think women might want reconstruction surgery?
3.	Have you heard anything negative about reconstruction surgery?
4.	What fears do they think women have about undergoing the surgery, or what factors stop or delay women from undergoing reconstruction surgery?
5.	What practical and emotional support, and information, do they think women need before reconstruction?
6.	What practical and emotional support, and information, do they think women need after the reconstruction procedure?
7.	If reconstruction were available in the United Kingdom, do you think women would want it?
8.	How do women feel about being involved in a research study?
B. 1^st^ National Stakeholder Event—Open Questions for Discussion Tables	
1.	What are your thoughts about whether reconstruction surgery and psychosexual therapy should be available in the United Kingdom?
2.	How do you think we should go about achieving this?
3.	What do you think the name of the project should be? At the moment it is called the RESTORE project.

*Note:* The table illustrates the discussion questions asked during the two workshop consultations (A), and the open questions for discussion tables at the 1st national stakeholder event held in February 2024 (B).

### 3.2 National Stakeholder Events

The first stakeholder event took place on ‘International Day for Zero Tolerance Against FGM’ and brought together multidisciplinary FGM experts and FGM survivors. It was advertised on Eventbrite as ‘a networking event’ for FGM survivors, professionals and stakeholders to discuss the lack of availability of reconstructive surgery in the United Kingdom and to hear their views on said topic (Appendix 3). Participants were reassured that the setting was a culturally sensitive safe space with counsellors on hand to provide support if anyone became distressed. A presentation on project goals from specialist midwives (J.A. and H.M.) was followed by a talk on ‘psychosexual therapy for FGM survivors’ presented by a psychosexual therapist (N.A.F.). Attendees were then asked to join one of three discussion tables to comment on the potential availability of reconstruction surgery and psychosexual therapy (Table 1B). Afterwards, participants were asked to complete a brief post‐event survey.

**Figure 1:**
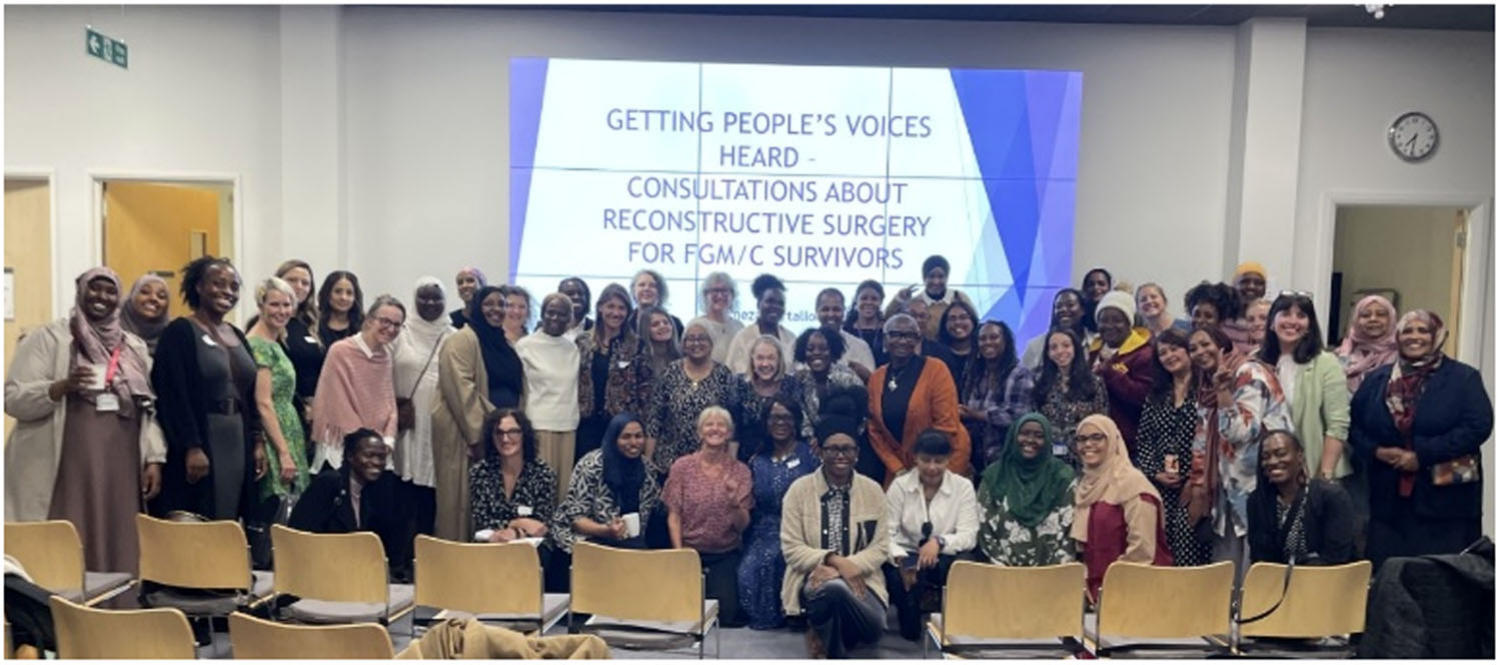
Attendees at the first national stakeholder event (Please note—Women at the event were invited to voluntarily join this photograph with the understanding that it would be used for dissemination purposes).

**Figure 2:**
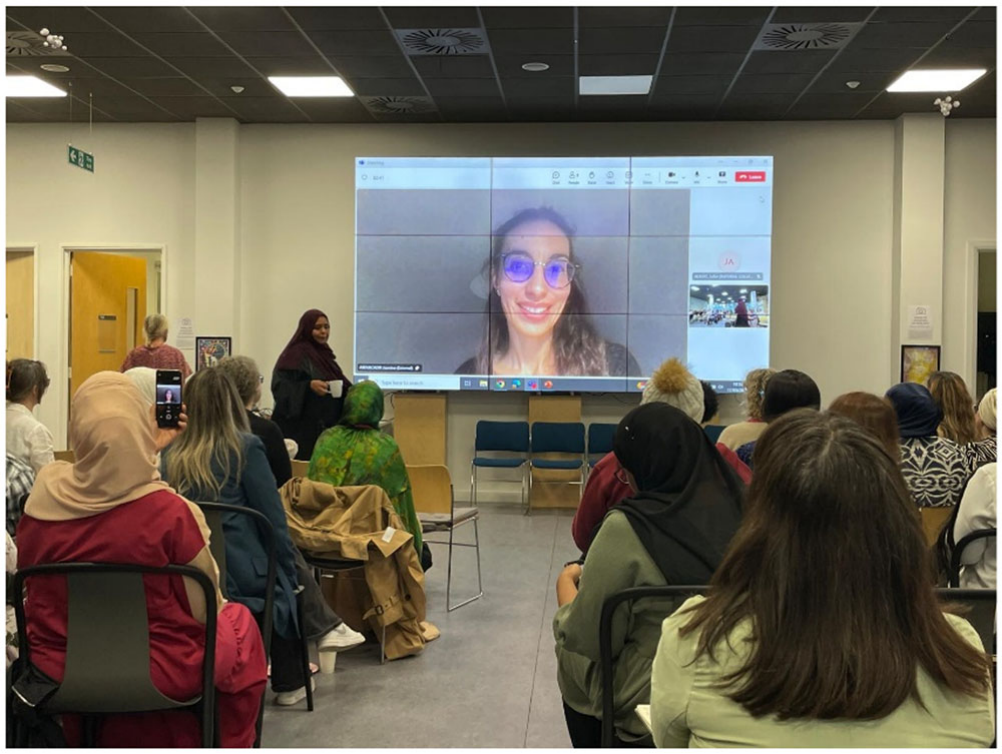
Virtual event speaker Consultant Gynaecologist Jasmine Abdulcadir shares her experience on FGM surgical reconstruction in Switzerland.

**Figure 3:**
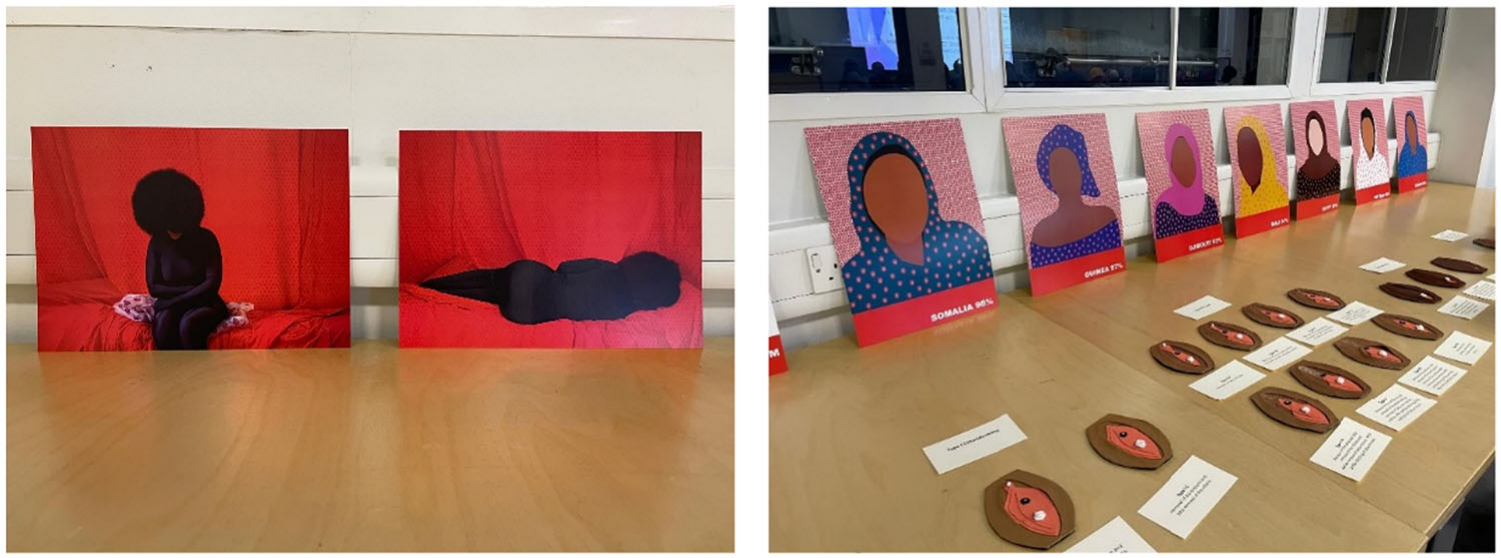
FGM Artwork from artist Aida Silvestri displayed at the 2nd national stakeholder event. Reproduced with the consent of the artist. More info on the artist available at www.aidasilvestri.com.

The second national stakeholder event took place 6 months later (Figure 1). Goals were to present the findings from the discussion workshops and scoping review publication and to hear from an existing reconstruction service in Europe. Consultant urogynaecologist (S.E.) gave an introductory presentation explaining why the project was set up and describing the work so far. Consultant plastic surgeon (A.A.) presented scoping review findings from the Collective's first publication [19]. Consultant gynaecologist (J.A.C.) from the HUG clinic, Geneva, joined virtually, sharing her experiences of leading an FGM reconstruction centre (Figure 2). Finally, psychotherapists (C.C. and N.K.) presented findings from the discussion workshops. FORWARD UK provided Arabic and Somali interpreters to increase accessibility and breadth of survivor input. We also had a Sudanese musical interlude, and artist Aida Silvestri displayed her FGM artwork (Figure 3).

### 3.3 Ethical Considerations

As a PPIE exercise, there was no requirement for ethical review, as engaging and involving members of the public and drawing on lived experience to inform aspects of research does not necessitate prior formal approval from an ethics committee in the United Kingdom [41]. However, we sought advice and clarification from our local ethics committee and adhered to recommended ethical standards throughout the process [42].

During the stakeholder event, we verbally announced that a voluntary group photo was to be taken for public dissemination and those who wanted to participate were welcome.

## 4 Results

### 4.1 Demographics of Attendees

The discussion workshops involved 11 FGM survivors, with a median age of 42.2 years (range 27–59). Countries of birth were Somalia (*n* = 3), Senegal (*n* = 1), Guinea (*n* = 1), Nigeria (*n* = 2), Saudi Arabia (*n* = 1), Sierra Leone (*n* = 2) and Uganda (*n* = 1) (Table 2). Fifty‐nine participants attended the first in‐person stakeholder event, of which 25 (33.9%) completed a post‐event survey (Table 3). The second event involved 83 attendees, and 29 (35%) completed a post‐event survey (Appendix 4). Details from the post‐event survey revealed, from the 29 respondents: attendees of the second event were mainly from London, with people travelling from Oxford, Birmingham, Bristol and Sussex. Eleven respondents disclosed they were either a survivor or from an FGM‐affected community. There were six doctors; eight allied health professionals (including midwives, trauma therapists and physiotherapists) and five described themselves as NGO/charity/campaigner‐activist/community‐champion. Ethnic backgrounds included Afghan (*n* = 1), mixed (*n* = 1), Arabic (*n* = 2), Black British Caribbean (*n* = 3), Southeast Asian (*n* = 1), white (*n* = 8) and African (*n* = 13). Ages ranged from 24 to 74 (average age 44).

**Table 2:** Demographic information for participants attending the two PPIE consultation workshops.

	GROUP 1						GROUP 2				
	Survivor 1	Survivor 2	Survivor 3	Survivor 4	Survivor 5	Survivor 6	Survivor 7	Survivor 8	Survivor 9	Survivor 10	Survivor 11
Country of birth	Somalia	Senegal	Guinea	Nigeria	Saudi Arabia	Nigeria	Sierra Leone	Somalia	Somalia	Sierra Leone	Uganda
Age	37	34	27	59	30	46	36	48	42	52	53
Age when FGM was carried out	4	Baby	7	6	13	7	7	8	4	10	12
FGM Type	III	II	II	Unsure	I	Unsure	Unsure	Unsure	II	II	IV
When heard of reconstruction	Years ago	10 years ago	Today	2019	2011	Midweek	2021	2 weeks ago	2 months	No response	5 years ago
How heard of it	Friends	Internet	N/A	Friend	Internet	Friends	Social media	Medical professional	Therapy group	Back on Track charity	Social Media
Do you know anyone who's had reconstruction	No	No	No	Yes	No	No	No	No	No	Yes	Yes
Should it be available in the United Kingdom	Yes	Yes	Yes	Yes	Yes	Yes	Yes	Yes	Yes	Yes	Yes

**Table 3:** Demographics of post‐event survey from the first national stakeholder event.

Demographic information	Number of respondents (%)
Total respondents	25
Ethnic background	White British: 6 (24%)
	White non‐British: 2 (8%)
	Black British: 2 (8%)
	Black non‐British: 13 (52%)
	Middle Eastern: 1 (4%)
	Asian: 1 (4%)
City	London: 21 (84%)
	Midlands: 1 (4%)
	Bristol: 1 (4%)
	Birmingham: 1 (4%)
	Leamington Spa: 1 (4%)
Age	Mean 37 ( ± 2.82)
	Median 37 (range 21–58)
Gender	Female 25 (100%)

### 4.2 Insights From the PPIE Discussion Workshops

#### 4.2.1 Why Do Women Seek Reconstructive Surgery?

Women shared their main motivations as being (in alphabetical order) body image, feeling ‘whole’, improving their womanhood in general; empowerment reasons; justice and equality; pain and discomfort; to improve relationship with partners; and to improve sexual function by increasing sexual pleasure or recovering feeling/sensation (Table 4).

**Table 4:** Motivations for seeking surgical reconstruction among FGM survivors.

‘Why do FGM women may want reconstructive surgery?’ Themes Identified, Quotes from the women (Group = G; Survivor = S).
Sexual Pleasure; Feeling/Sensation G1 S1: ‘I wish I could have great sex and I could orgasm’ G1 S2: ‘Sex has never been good. Having that piece of respect might change something’ G1 S3: ‘I know people who are cut, and they really enjoy sex. So that's punishment to me. That's punishment. It certainly seems, through the media and films and everybody makes it look like everybody loves it, you know. Everybody's having a great time’ G1 S4: ‘Pleasure is an important thing, and even self‐pleasure that's not something that is possible (at the moment). Because being by yourself and experimenting with self‐pleasure does nothing, at least for me’ G1 S5: ‘A sense of what is my real pleasure is, because for so long I've been pretending. What's real? What's made up? I honestly couldn't tell you. So, having that reconnection to my body in that way where I have like an internal access to my own truth about my pleasure, rather than looking to other people or looking to literature or reading about it instead of feeling it intimately in my own body, having that direct line to my own pleasure would be great.’ G1 S6: ‘It's a part of my life that I want to experience, something great, and feel whatever I need to feel. So it's important to have that option (clitoral reconstruction)’ G1 S7: ‘my friend did it to improve her sexual pleasure.’ G2 S11: ‘My own problem is that I have, like, like feeling. Feeling problem. It's not easy for me to just catch up feeling just for sex. Just like that. Carelessly. It's not easy. Just like everything is gone. Just like I said, it's clean so the feeling is slow. And that's why I said if I have the power, I will fix it back.’ G2 S11: ‘I wish I could have my feelings back, for me that's very important.’
Pain and Discomfort G1 S2: ‘Or it might be because they've got long‐term pain. I had this cyst 3‐4 centimetres so I couldn't wear underwear or trousers, so even walking was painful. I'm still suffering and my GP prescribes me antibiotics. I had the surgery in March 2022 to remove the cysts and that's why my husband left me. Now I'm still having infections on the clitoris and I returned to my GP who referred me back to the surgeons and they said there is a chance that the cyst came back again. So I have to live with that and I have to live with the fact that I don't have any more clitoris and I have damaged nerves that's why I still have the pain and they gave me anaesthetic gels that I'm using because anytime I wear underwear I can feel pain.’ G1 S7: ‘Some people have pain and discomfort all the time’ G2 S11: ‘So this pain has been going on for years. I've done all sorts of tests, but they cannot find out what's wrong with me’
Feeling Different/Wanting To Be ‘Whole’ G1 S1: ‘I feel like I'm missing something’ G1 S2: ‘I'm the only one who's different. For me to have that (clitoris) back, it might feel like, okay, I'm part of the group now. So, maybe when you're confident and kind of think you look at yourself in the mirror and think I'm back to normal’ G1 S4: ‘In terms of reconstruction, I think it's important to have the option of having the reconstruction, without it that person's going to suffer for the rest of her life, or not feel a whole or fulfilled.’ G1 S7: ‘my friend also wanted to feel whole/normal again.’
Body Image G1 S4: ‘I'm constantly conscious of the difference between my body and the standard body. I know there's no standard body, but I know that I'm missing something that's like core and can't be replaced by anything else. So it feels like it's integral that I have it back to feel whole in some way, and that affects everything like body confidence, like feeling comfortable in your own body, but then also with a partner. I think it leads to a lot of insecurities that then bleed out into other things beyond like, you know, anything to do with your body or with sex. So, if you are not peaceful with your own body, you cannot be comfortable. You have to be peaceful with your own self.’
Womanhood G1 S2: ‘Since I've turned 34, I've wanted my womanhood back. I obviously feel the FGM has a lot to do with that, so I feel like, you know, fake it till you make it kind of thing. I feel like I have to walk in a certain way, or hold myself up to remind myself that I am a woman, although these horrible things have happened to me. I think it's really important to have that option, to have the reconstruction, if I wanted to feel okay maybe this is the next step.’ G1 S5: ‘I've heard so many different stories from women with FGM who, on their wedding night, had to go to a clinic or hospital and then straight away to have sexual relationship with a man, and then get ready to have kids. They hate sex from day one. First you had FGM, then they cut you to have sex with a man who doesn't care about your feelings, your pain. You didn't pay for the bleeding. They do all of that to treat you like a toy, and then when you get freedom, you don't feel like a woman, you're just a thing, that society decided to treat you like a thing. You can blame the family or the society, but we forget about that woman who had the FGM’ G2 S11: ‘When I look at myself, it's awful. I don't look like other women.’
Empowerment G1 S2: ‘It's about getting your power back, understanding yourself, knowing your body, and knowing what you need to feel pleasure within or outside of a relationship, which is not available at all to a lot of people I know’ G1 S5: ‘when have I been thinking, oh, tonight I'm going to have sex? It was just him wanting it. And it's like, why not get your power back? Maybe you want it as well. Maybe it could be a power, kind of like owning yourself again and finding yourself’ G1 S6: ‘They treat you like a toy, and then you don't feel like a woman, you're just a thing, even society decided to treat you like a thing’ G2 S11: ‘Self esteem…. Me, I'm not scared. It's something that I would, I would say yes to right away. Yeah. I'm like when they told me about this meeting I was like ‘Oh, wow, dream comes true.”’
Relationship G1 S2: ‘It's also a big reason why a lot of women don't end up having partners or seeking partners or surviving long in relationships. It's because of that fear of not living up to a particular standard or normality or feeling what they're supposed to feel during sex or just looking normal, you know what I mean? It puts a lot of pressure on relationships a lot of the time, like a lot of psychological pressure on the woman as well and can lead to the breakdown of a lot of relationships. I think it's also making sure that you can be in a relationship long term or not have that fear of not being a normal partner.’ G1 S3: ‘The man sees you as the one who's always been laying down doing nothing or jumping from wherever, so you end up being a chore instead of being something enjoyable’ G1 S4: ‘it's like you're being punished, by having FGM, and then you're being punished by your partner for not trying. I mean, it's weird how you get blamed for something that was done to you, and then it's your fault that you're not enjoying sex, or event being told “you don't want me” or “I'm going to go and cheat on you, or get a second wife.”’ G2 S9: ‘every time I have a relationship. like, my first one—I was 16, the boyfriend and he lasted like one month. I don't feel anything.’ G2 S9: ‘Because now, like, you go to a man who is not from your culture. And then; “oh, what is this?“ They get scared because you're different.’ G2 S11: ‘They say “You sleep and you don't feel anything, Get out”. “You don't love me, or you don't want me.”’ G2 S11: ‘you met a guy from our place as soon as I met you at the party or wherever, they start talking to you on the phone and say too: “Oh! I hope you haven't chopped off because I want to [unclear] to have it. And I still don't say anything. And then finally if we fall in love, and we have sex, and they notice. They gone.”’
Justice/Equality G1 S3: ‘Isn't it ironic that men are allowed to get vaginas? You can have feminising surgery. For a man to say “I want to feel like a woman” is possible, but we (FGM/C survivors) are feeling the same thing and we are not being heard. We don't feel like every other woman. We're like, there's something missing. So who's got more rights? But as a man you choose to have the option compared to someone who didn't even have any option at all, who had something done to them and they didn't ask for, and is affecting them in a very weird, drastic, crazy way. FGM/C it's affecting women. It can affect their family, their relationship, their mental health. For us to be begging to have the free access to reconstruction while the same women in other countries would be getting since years and years. I feel like it's different for us. I guess we can't complain because we are foreigners.’ G1 S5: ‘I was going to say, beyond just sexual pleasure and body confidence and image and stuff, I think it's just a sense of justice. It just feels so unjust that something was done to you before you had any understanding of it and that it has such a massive effect on every single aspect of your life. A lot of the time it just feels hopeless or like there's nothing that you can do about it and you just have to live with this condition that you never chose for yourself. So I think also reconstruction is a way to get justice, because you can't go back in time. You can't undo, like I can't undo my family's views on these things. Like they're set in their ways. I don't necessarily want them to be punished for it because they learned it from someone else and they did it thinking that it was good for me. So it didn't come from a bad place, but it has bad consequences. So I think about reconstruction as a sense of justice that's not harming anyone, but that's actually helping me, I think it is super important.’ G2 S12: ‘if this operation can be done in the UK, we're going to be very grateful.’

*Note:* This table reports themes emerged during the first and second consultation workshops, with verbatim quotes from the women.

The following quotes are taken from the women in the discussion workshops, referenced with Group (G1)/Group (G2)

*G1 S2: ‘It's about getting your power back, understanding yourself, knowing your body, and knowing to feel pleasure within or outside of a relationship’*

*G1 S3: ‘It looks from the media and society that everybody love sex. Everybody's having a great time, except me’*.

*G1 S4: ‘I'm constantly conscious of the difference between my body and the standard body. I know there's no standard body, but I know that I'm missing something that's like core*.’

*G2 S8: ‘The scar tissue is the main reason for pain when I have sex. I have done many tests, at the NHS they told me that I have pain because of my scar tissue that hurts during penetration. Then, why don't they fix my scar?’*

*G2 S9: ‘Because you go to a man who is not from your culture. And then they say; “oh, what is this?” They get scared because you're different.’*

*G2 S11: ‘They say “You sleep or what, you don't feel anything, Get out. You don't love me, or you don't want me…. “You meet a guy and then finally we fall in love, and we have sex, and they notice that you have been cut. They're gone!”’*

#### 4.2.2 Where Do Women With FGM Find Out About Reconstruction?

Two women had not heard of reconstruction surgery before the workshop. The others had heard of it mainly through social media or talking to friends (Table 5).

*G1 S2: ‘I go through intense Googling on FGM every now and then. I'm like, what is out there? What can help me? I need help. Because I don't really talk about it with anyone. My only access to understanding other women's experiences is by going online. So I read a lot of posts from women who've gone through reconstruction. That's my only access to what reconstruction is.’*

**Table 5:** FGM survivors insights and reflections regarding a potential future service configuration.

1. Where Do Women With FGM/C Currently Find Out About Reconstruction?
Online/Social Media G1 s1: ‘online research, mostly on reddit.’ G1 s2: ‘i go through intense googling on fgm every now and then. I'm like, what is out there? What can help me? I need help. Because i don't really talk about it with anyone. My friends don't really know. I don't really talk about it. I think my only access to understanding other women's experiences is by going online, like searching for stuff. So i read a lot of reddit posts from women who've gone through reconstruction. That's my only access to what reconstruction is. To be honest, a lot of them are very positive. The only negative thing i've heard is that it didn't have the effect that she thought it would have. And that's been a big frustration and disappointment.’ G1 s3: ‘online for me. At that time i didn't even know i had fgm. It was just curiosity. I heard people talking about it. And they were actually talking about a doctor that does it in france. Famous name. I don't remember the name. (Dr Foldes). Yes, that's the one. And there's a lady that was telling other ladies to go because she did it and she knows a lot of people that have it. They were just saying good things. I didn't hear anything negative about it’. G1 s5: ‘i actually emailed dr. Dey because i saw their tiktok talking about reconstruction in Germany. And there was nothing here in the uk. So i emailed them’ Talking to Someone Who Has Had It Previously G1 s6: ‘as a woman, sometimes you just discuss things. The first time i heard about reconstruction, it was from one of my neighbours who told me. Our generation, i think over 30, if you were born in somalia, you are most likely to have fgm. She just mentioned something to do with getting your clitoris back. That's all i remember. I didn't know whether it was available. She was like, oh my god, you know you can get your clitoris back again.’
2. Who Do FGM/C Survivors Talk to About Reconstruction or FGM/C in General?
Nobody G1 S3: ‘I haven't really told anyone, in terms of my community or my friends that I've been through FGM. It's not something that's really talked about in my family.’ G1 S5: ‘I've never spoken to anyone. In my family, it's not talked about, it's just not something that you say. They know, obviously they did it to you, but you don't really talk about it, and as I had my daughter, I started thinking, actually I've been through a traumatic thing, let me deal with it, because it was impacting my life, and everything's followed me from when I was three and a half until today.’ G1 S7: ‘Only to someone I trust. This is an intimate subject. I need a safe space. I don't want shock or pity.’ G2 10: ‘I do not even discuss it with my culture. I say I did not have the cut done, because it is difficult for me to have the conversation’ G2 S12: ‘it's very, very embarrassing. I know lots of people from my culture now have done it, but we don't be talking about it. Oh, why do I have no feeling? What about you? Did you get chopped up? I said, no, I tell lies because I just don't want to discuss it with anybody.’ Family Member/Friends G1 S1: ‘My family and my partner.’ G1 S2: ‘My mom, my sisters, they also experience FGM.’ G2 S10: ‘Some of my friends’ Healthcare Providers G2 S8: ‘GP. The community is still promoting FGM, they believe like ‘it has to be done’, so I speak to other people’ G2 S9: ‘Professionals only. If you talk to someone who strongly support and believe in it, they won't listen’ G2 S12: ‘It's better to speak to a professional; it is better than their family members. They believe in FGM.’
What Can Stop or Postpone a Woman With FGM/V From Undergoing Reconstructive Surgery?
Negative Beliefs G1 S5: ‘When I heard about reconstruction I was like, no. I'm so scared. I don't want to do it. What if I don't get a husband and then I feel like I'm going to have sex all the time?’ G1 S7: ‘Having surgery could also trigger memories. It might also make you feel that you don't belong i.e. you're not from here. As people are prejudiced’ G2 S9: ‘I'm very, very coward. Yeah, I wanna do it, but my mind is like this. Yes, my mind is not steady, but I really need it. That's my problem, why I'm scared.’ G2 S9: ‘If I do the surgery, if it's going to be like the same pain I received last time—so, that's my fear’ G2 S9: ‘Because some girl bleed to death…. That makes me scared more. The bleeding, yeah.’ Recovery Time in Relation to Family Care G1 S3: ‘if you're looking after kids you don't want to take a break if you're not able to do anything after surgery, so you don't know what's going to happen, are you going to be able to care for your kids after the surgery and be off work, how long would it take? So somebody might wait to have it until things in their life are more sorted or maybe feel like they can't ever get it because their life will never be calm enough that they can recover.’ G1 S7: ‘A lot of counselling is needed. It a big decision. It's risky and bring up painful memories and may ruffle people's feathers’ G2 S10: ‘Complications, such as bleeding but I know they do blood test and all the check like for cesarian section.’
What Fears Can a Woman With FGM/C Face About Undergoing Reconstructive Surgery?
Judgement and Exclusion From Their Community G1 S1: ‘I think some women as well, like when they get, like the one that I had (defibulation), when they get opened or whatever. Or like they had complications and then they tell their friends and then their friends say stuff. So it's kind of like there's a small community that talk about it. So, yeah, especially in the Somali community. They don't get married and they see you're different from the ones they knew, so they don't understand themselves, for them to even help you. So, you know, social media, now if you do anything like this, people have to be very careful. Even if you go to a party, maybe you want to take a drink, but everything is social media.’ G1 S2: ‘There's already shame and judgment around having gone through FGM and will there be the same shame and judgment around getting a reconstruction?’ G1 S6: ‘I think it's a tough one because you don't want to do it in a way that would put women's safety in danger within their own community. I just want to stay safe in my community rather than explore this thing that might put that in jeopardy. And the other thing is you also don't want to be vilified by your community. And sometimes that can also stop a lot of women, feeling guilty in some way, coming to terms with the fact that their community or society did this to them, you know. So I think it's a tough one.’ G1 S7: ‘How partners and husband might react if you get reconstruction. Wanting to reverse it is rejecting your culture. Women not conforming to husband's ideas. Your body isn't your own. Man may ask “Why do women want sexual pleasure. Sex is for men to enjoy. Why do you want to discuss such personal things … there may be cultural resistance.”’ G1 S2: ‘I've already been through something traumatic then I go through this thing without a real community around and how do I deal with whatever is thrown at me?’ G1 S7: ‘It may ruffle people's feathers’ Emotional Aspects G1 S1: ‘I think, for me, there has to be support before and after, because the idea of having support until the point of reconstruction and then kind of just being left there sounds terrifying, because there may come a lot of changes with that, I imagine, so I think that is one thing’ G1 S3: ‘The pain that comes with it and how it's ruined your life. If you do reconstruction, if it does restore anything? it's a bit like we've kind of put it away in a box, possibly sadness and pain or like anger at family, like all of that well I think will explode’ G1 S5: ‘I think also it triggers a lot of things like I'm just thinking about the idea of a doctor being down there changing things. I'm just transported back, because I was a bit older when I got FGM, I was 13, so I remember everything, I didn't have anaesthetic, nothing, so I literally remember it, and I think that it is one of the most traumatic things that has happened to me. So to be in a similar kind of relationship to a doctor would trigger so many memories that I've really really tried to repress. So I think it's also situational like that process where does it take me mentally? So you're going through the same process again like the second time, obviously it's different but I think it can activate a lot of memories.’ G1 S6: ‘To go through such a big change alone, because FGM already feels such like such an isolating experience, so will the reconstruction be the same also in some way.’ G1 S7: ‘Having surgery could also trigger painful memories. It might also make you feel that you don't belong i.e. you're not from here. As people are prejudiced.’ Complications or Risks of Surgery G1 S1: ‘Maybe fear like scared that they might have complications’ G1 S3: ‘Maybe the complications’ G2 S11: ‘It can trigger memories from the past.’ G2 S11: ‘The scar tissue for example. I would like to know pros and cons of surgery, including surgery. I would want to know about potential complications.’ G2 S8: ‘Is this a major operation or is it a very simple region?’
What practical and emotional support and information should women have before reconstructive surgery:
Education About Body Functionality Related to Sexuality G1 S2: ‘I would say that if I had the surgery, I would want to learn about my femininity, how I connect with my body. Because I think that part of it is just so shut down from everything. You never really get to feel like a feminine woman who feels just fulfilled within herself and knows her body and her sexual pleasure. But now, once I've had the surgery, now that I've got this, now I'm part of the group now, how do I belong? I think sexual education is so geared towards women who haven't had FGM. Because every time I like try to build this mythical relationship to my sexuality, whatever that is, like the advice just doesn't apply to me. I'm like, this will not work. And I know it because I've tried it a million times. So I think sexual education that is actually geared towards women who've been through FGM. As standard sexual education has lot of gaps that don't account for women who've been through something like FGM. So I think sexual education that actually takes our experiences and difficulties into account. Yeah, because sexual education now just mentions FGM as a footnote, like some women can't do this, moving on. You know? and it tends to be, oh, well. And it's even more alienating because you're like, oh, I'm coming here for help to feel more connected to myself. I feel more disconnected and more like made to feel like I'm different and not normal and all of these things. So yeah. This is more about counselling.’ G1 S7: ‘Psychological and professional support. Frank and honest. Information is power. It may trigger memories and there maybe a lot of shame about it. Someone needs to check in on you and how you're doing. I think at least one psychosexual session should be compulsory and then optional. So women understand why they want it and to explore the psychological and physical impact of having surgery. Not just have surgery and go.’ Couple and Family Support G1 S1: ‘Maybe say if the person's already in a relationship and they have everything, but the partner wasn't involved in all that process, how do you get them along just to help the woman to go through that journey and go back, enjoying their sexuality kind of thing in general? Yeah. A couple. Just how to get the partner involved in that. So support‐wise or sexuality‐wise? In general, I don't know. Because if, let's say, if someone was in a really long‐term relationship, and then the aim for you to have the surgery is in a positive way, but that man you're with without any help and advice from professional doesn't even know how to help you go through that journey. They might mess it up for you.’ G2 S3: ‘your family support, just to kind of get the gist of what's happening in that woman's personal life. Because I guess you can't hide it. No, you need help. You need recovery time and support. So I think that's quite important. The biggest fear I have, even though I don't really have a lot of family around me, is the shame that I would have around my community. That I've…. Just to do that would be so courageous and so scary, I can imagine. Would you not tell anyone? Like, it's just so scary. But so I think putting in place safety measures for the woman to actually be OK after the surgery. And what that looks like before and…. It's a really important fear thing, because it's, you know, we don't know whether… Would there be some sort of backlash? Yes. Like, you know, should women be doing that for themselves, taking the power?’ Safeguarding G1 S4: ‘I would say, like, more information in terms of, say you had a woman who's very close to her family. They're in her life. You know, they're in her house all the time. And there are a lot of women like that. And she wanted to have the surgery, but she wasn't going to tell anyone. Like, that to be discussed, you know, could she be, like, maybe unsafe if she was to have the surgery without their permission or her husband's permission or her family. What would be put in place for that?’ G1 S6: ‘What about protection? Because some people, depending on the community that they're going to be in, they might get really negative…. It might have a really negative impact for her to go through. Even though she really wanted to do it, but she wanted to do it in a way nobody around her would know, because once they find out, then she'll be in big trouble. Would there be another way to help that person go through the process, finish it, without anybody that could harm her from finding out? Some communities will be like, the woman shouldn't be allowed in there. For them to know that you went through that, they're just going to think you're going to be affecting all other women. Yeah. It can be a family thing, but even the husband's side. And you mentioned shame.’ Management of Expectation G1 S5: ‘I really feel that the whole point of that support before making the decision, is managing expectations around what will come out of the reconstruction, and if it's something that I want, and also what are the risks, like emotional and physical that come with it, and am I willing to go through that for something that may or may not improve the things that I want improved, you know so, it's kind of extensive consultation because as you're saying, it's an individual. You all may have similarities but maybe differences. So that's extensive consultation taking into account your own personal situation and am I in the right position mentally for a process like this? do I have the support around me to be able to do it?’ G2 S10: ‘opt to live a normal life like other women like the way God created you’ G2 S10: ‘So, what are you going to like or replace to fix this then that is gone?’ G2 S8: ‘I would like to know how the vulva it would look like, how open it would be, like a very big hole or not.’ G2 S10: ‘I'd like to know if I have surgery if I can get, my feelings back. That is for me the most important.’ Counselling G2 S11: ‘Professional counselling just to tell you that, yes, you're going to be okay. Yeah, just professional counselling, so that when you go for the surgery, you think: “I am, I'm well and I'm going to come out with it.”’ G2 S10: ‘counselling that someone wants to tell you that you, when there's no hope for a change, it's to tell you that “Oh, sorry. Just live with a positive attitude.” But still, I don't think anyone can say that I would want to live the same way, because even the actual, looking at it like it's, like, “no, this is not nice this is awful.”’ G2 S11: ‘Trauma management.’ G2 S11: ‘When they go in operation out, they need someone to talk about their feelings and yes, they need it. Because they have a new system in their body, and they open it, they need to talk about how they feel.’ G2 S11: ‘I think it starts with the mind, you know—“*what's on your body? How is it affecting your mind?”* If your mind is sick because of what you have and what you're going through, and there is…. They, they show to you that there is this option of bringing back your mind to normal and feeling good, I don't think someone can say that, can have a negative attitude about it. Personally, no.’ Contact With Women Who've Had Reconstruction G1 S7: ‘Peer support from women who've already had it would be good.’
Where do women with FGM/C would like to find out that there is reconstructive service available?
Healthcare Providers (e.g., GPs, midwifes and sexual health clinics) G1 S1: ‘I think it's mainly GPs because everyone's got access to GPs in this country. So if you want to get the word out, it should be given out by GPs. Because your GP would know if you had FGM or not and then for them to give you that information.’ G1 S4: ‘if you just heard about it, it doesn't have to be social media. And then you might go to your GP for more information, but you know, to get it out there.’ G1 S7: ‘In GP surgeries, sexual health clinics, when having smear test.’ G2 S11: ‘I think, some other most important ones would be midwives. Yes. Because our community don't talk about these things, so when a baby goes to the midwife, that is the most important part, because they will talk to them and explain to them. And that is the most important because (in) our culture, they don't talk about these things.’ G2 S12: ‘I suppose the GPs as well because you, for example, if you're your younger woman, you might not be in contact with the midwife for example.’ G2 S11: ‘Midwives, doctors.’ Universities, FGM Organisations and Social Media G1 S1: ‘Awareness campaigns maybe? Maybe going through universities, having posters around. I know it's not something that everyone would be needing it, it's mainly from like the African community. So how do we get to them? I think social media makes sense also because a lot of people keep it hidden and don't say anything. So it's easier, if I saw like a physical campaign somewhere, like a stall, I wouldn't go up, you know, because of the shame associated with it and I just want to kind of ignore it, especially if it was in a community setting, like let's say in my community, I wouldn't go up to something like that in front of people, but if it was on my phone, if it was something anonymous in some way, like I would click and you know, like look at it a bit more, or leaflets. Community centres as well, targeting Somali community centres, different ethnic minorities. FGM organisations, putting the word out there, maybe holding events or something.’ Places of Worship G1 S3: ‘Mosques or churches could work. I don't, I would not recommend that, but it's worth trying to go there and see how they will respond to it. Because the communities we're talking about, are communities that actually listen to the priests or imams a lot. For them to be a part of this would be a really big thing. So to get the people to be more educated about it, especially men and their families. Like if a man says, no one would ever do this to my daughter, it would never happen. But if the woman says it, no one cares. It just seems like it just starts from them. So for those communities, it's how we have been raised. Churches and mosques actually go’ Word of Mouth G2 S12: ‘I know a lot of people I can tell them they will be very pleased, to do it.’ G2 S9: ‘Our friends are victims’

*Note:* The table reports the themes that emerged during the first and second consultation workshops, with verbatim quotes from the women (Group = G; Survivors = S).

#### 4.2.3 Who Do Women Talk About Reconstruction?

Some of the women described talking to someone they trust (e.g., family members who had experienced FGM, friends and/or partners). Others reported they had not discussed surgery or FGM with anyone before. There was discussion about the silence surrounding FGM and that it is considered a ‘taboo’ topic (Table 5).

*G1 S7: ‘Only to someone I trust. This is an intimate subject. I need a safe space. I don't want shock or pity’*

#### 4.2.4 What Might Be Reasons for Not Having or Delaying Surgery?

The following aspects might stop or postpone a woman from undergoing reconstructive surgery: misconceptions (e.g., fear of becoming constantly aroused); emotional aspects and trauma (e.g., triggering memories of when FGM was performed); and recovery time that would not be compatible with family caring responsibilities. They also described safeguarding concerns related to judgement/exclusion from their community, negative reactions from partners/husbands and complications after surgery (Table 5).

*G1 S5: ‘When I heard about reconstruction I was like, no. I'm so scared. I don't want to do it. What if I don't get a husband and then I feel like I'm going to have sex all the time?’*

*G1 S7: ‘How partners and husband might react if you get reconstruction. Wanting to reverse it is rejecting your culture. Women not conforming to husband's ideas. Your body isn't your own. Man may ask “Why do women want sexual pleasure. Sex is for men to enjoy…”’*

*G2 S9: ‘If I do the surgery, if it's going to be like the same pain I received last time—so, that's my fear’*

#### 4.2.5 What Practical and Emotional Support/Information Would Women Require Before Surgery?

Responses included: education about body functionality related to sexuality, psychosexual counselling, psychological support, particularly regarding trauma, couple/family support, safeguarding, management of expectations and peer support from women who already underwent reconstruction (Table 5).

*G1 S2: ‘if I had the surgery, I would want to learn about my femininity, how I connect with my body. Because I think that part of it is just so shut down from everything. You never really get to feel like a feminine woman who feels just fulfilled within herself and knows her body and her sexual pleasure. But now, once I've had the surgery, now that I've got this, now I'm part of the group, now I belong’*

*G1 S6: ‘What about protection? Because some people, depending on the community that they're going to be in, they might get really negative…. It might have a really negative impact for her to go through. Even though she really wanted to do it, but she wanted to do it in a way nobody around her would know, because once they find out, then she'll be in big trouble*.’

*G2 S11: ‘I think it starts with the mind, you know—“what's on your body? How is it affecting your mind?” If your mind is sick because of what you have and what you're going through…. They, they show to you that there is this option of bringing back your mind to normal and feeling good.’*

#### 4.2.6 Where Would Women Like to Find Out About Reconstructive Surgery Services?

Women suggested various information sources, such as GP surgeries, sexual health clinics, when having smear tests, local community centres, universities, FGM charity/NGO organisations, social media, places of worship (i.e., churches and mosques) and other women (Table 5).

#### 4.2.7 Other Insights

Other responses demonstrated a lack of knowledge of FGM types or clitoral anatomy and a lack of information regarding access to and availability of FGM specialist services.

*G1 S4: ‘I never knew I had a clitoris down there, or whatever, and now I know, and I'm like, I have one! That's great today! Especially, however, the doctor told me you don't have a clitoris anymore. I've had to live with that in my head’*

*G2 S9: ‘Women who've been through FGM just feel like there's nothing out there for them.’…‘I think a lot of people would be up for it, (reconstruction) they just don't know. You know how many years I've looked for a counselling service, I wanted it, but I just didn't know what was there’*.

### 4.3 Insights From National Stakeholder Events

Many similar themes were discussed during the stakeholder events. Participants engaged in discussions regarding sexuality, including misconceptions and a lack of understanding about female genitalia and sexual functioning. Most attendees said they believed reconstruction should be available in the United Kingdom. Their reasons included: this is an unmet need, and women are forced to travel abroad at high costs they cannot afford. Only one participant disagreed, however, citing the limited clinical evidence. FGM survivors also strongly supported suggestions that psychosexual therapy should be integrated within a holistic service with pre‐ and post‐surgery support, education about body functionality, and trauma counselling.

Event participants identified a need for psychosexual education for survivors, healthcare professionals and the general population—for example, we found it is often believed (mistakenly) that FGM survivors are unable to experience orgasm and that their clitoris has been completely removed. In addition, some FGM survivors knew of specialist services, but many lacked information about these.

Another important finding was that some attendees disliked the project name (formerly called ‘Restore’). The name was subsequently changed to ACERS_UK in response to this concern.

There was consensus that there should be further such events, perhaps online, so that more people could attend and that the project's work should continue. Attendees appreciated the simultaneous translation and particularly valued the live Q&A sessions with clinician J.A.C. Overall, there was enthusiastic feedback from attendees towards the stakeholder events (Appendix 4). The following quotes are taken from the post‐event survey and referenced E1/E2

*E2 ‘Interpreters helps survivors feel included rather than always us speaking out for them. And I think it acknowledges that women here in the UK are sometimes still struggling with language.’*

NGO/charity worker

*E1 ‘The honest truth about the procedure and knowing I can have the reconstruction’*

FGM survivor

*E2 ‘Having Jasmine, a clinician who's been doing this for 10 years. She has data as well. It's important to share this evidence—it's not something that we're just imagining’.*

FGM midwife

*E2 ‘It gives hope for the survivors who can see the other end, that change can happen’.*

Doctor

*E2* ‘*This event was inspirational, and it was so amazing to be in the room’*

Allied health professional

## 5 Discussion

This PPIE work combined small discussion workshops and national stakeholder events, aiming to inform future research and service development related to genital reconstructive surgery for FGM survivors. Events were co‐produced with input from the entire ACERS team, representing multidisciplinary expertise. The PPIE consultations highlighted many knowledge deficits. Feedback from the events is discussed below.

### 5.1 Reflections Upon Research and Service Development

We learned that FGM survivors living in the United Kingdom might seek reconstruction surgery to address specific physical and psychological needs, such as general genital pain, pain during intercourse, relationship problems related to sexuality, feeling whole and body image. Similar insights were identified from both workshops and align with similar studies conducted in European countries [43, 44, 45], but to the authors' knowledge, have not been explored with UK survivors before. A previous (2015) UK‐based PPIE consultation with a broad selection of representatives from different FGM‐affected communities [28] similarly identified the need for services that address both women's and men's psychosexual health and involve affected communities in service design. Surprisingly, reconstructive surgery was not mentioned in that consultation, suggesting a need for further investigation into why more women are seeking this procedure, potentially influenced by social media and increased awareness.

Another key insight from this study was the need for improved psychosexual knowledge, including addressing misconceptions about sexuality and FGM. Research examining the anatomy of the clitoris has proven that, in many cases, only the external tip (the glans which is approximately 10% of the whole clitoris) may be removed during FGM, as the main body of the clitoris mostly remains buried beneath the external genitalia. Despite this information being available for several years, many women and healthcare professionals remain unaware of this anatomical reality, and a gap in knowledge is clear. Furthermore, sexual pleasure is a highly complex issue [44, 46]. Studies demonstrate that many women with FGM can experience orgasm, and many women who have not experienced FGM report being unable to orgasm [47]. This highlights the need to integrate psychosexual education and therapy to address sexual dysfunction and to ensure we avoid or mitigate unrealistic expectations when exploring reconstructive surgery.

Stakeholders overwhelmingly agreed that reconstruction should be made available and survivors should co‐design future services.

Other issues that emerged relate to the principles of equity and justice. We found that FGM survivors experience a sense of double injustice—firstly, they were forced into having FGM, and secondly, they are unable to access a health intervention that women living in many other countries can. Moreover, some women voiced their anger at the paradox that vulval reconstruction surgery is available in the United Kingdom for some health conditions, but not for FGM. For example, the NHS commissions feminising genital surgery, involving the creation of a clitoral glans, vulva and inner and outer labia, despite limited evidence on the subject. Furthermore, vulval reconstruction techniques are available to vulval cancer survivors after vulvectomy and to treat lichen sclerosus. This unequal access to genital surgery appears to be a significant health inequity and appears to be in direct contrast with NHS England recommendations stating: ‘Commissioners must understand local population needs in relation to FGM’ … and ‘consider services to support non‐pregnant women and girls wanting support and/or treatment’ [48].

Another important finding was the need to develop a glossary of research language to be used (and translated) for future PPIE work, which will continuously evolve. For example, recognition that the word ‘reconstruction’ is problematic, implying that FGM survivors are in a deficit state.

### 5.2 Reflections Upon PPIE

Since many women are hesitant to talk openly about their experiences, a culturally sensitive and inclusive PPIE approach is essential. For example, on one discussion table, a health professional repeatedly referred to women with FGM as ‘*victims’*, until a woman spoke up and said, ‘*I don't wish to be referred to as a victim, I'm a survivor’*. In the future, we acknowledge the need to set explicit ground rules to ensure culturally sensitive language is adopted and agreed upon by all those present and to request that no one dominates discussions.

This PPIE provides compelling evidence of the need for more research in the field, ideally a clinical trial. Its insights also inform potential future aspects of research design, such as the most appropriate associated care pathway. Overall, FGM survivors and stakeholders considered that services for surgical reconstruction should be explored in the United Kingdom.

Overall, this PPIE work provided valuable reflections that could help improve engagement and services in this arena and provides essential insights to inform future research. This will ensure that patient‐centred care is at the core of future reconstructive services and that we listen to the concerns and perspectives of women living with FGM.

### 5.3 Strengths and Limitations

This study presents a number of strengths. Firstly, this exercise demonstrated that, with the right approach, it is possible to engage ‘minoritised communities/individuals from the global majority’ on sensitive topics, despite often being labelled as ‘hard to reach’ [49]. It is also important to recognise the concept of these communities being ‘hard to hear’ when it comes to listening to survivor voices or experiences. We found that all stakeholder events were met with great enthusiasm, and many women expressed interest in future involvement. Successful PPIE for clinical research involves multiple factors. Barry et al. described participatory research as blurring the line between ‘researcher’ and ‘subjects,’ emphasising shared knowledge pursuit and societal change [50]. ACERS members adopted a participatory partnership model that includes FGM survivors as experts, ensuring co‐design and collaboration from the start [51].

Secondly, the United Kingdom's National Institute for Health Research (NIHR) provides crucial infrastructure and strategic assistance, establishing a framework wherein PPIE is regarded as an essential component of all publicly financed research [27]. This study adhered to the GRIPP2 checklist principles, ensuring quality, accountability, transparency and evidence‐based practice [32, 33] and aligned with NIHR INVOLVE's co‐production principles: shared power, diverse perspectives, mutual respect, reciprocity and relationship‐building [30]. Despite its strengths, this enquiry also has limitations. Our primary aim was to hear from FGM survivors about their attitudes towards reconstruction, rather than addressing broader issues related to FGM. To maintain a focus on reconstruction and to orient participants to the topic, the workshop activities posed specific questions that may have been leading. Likewise, we prefaced workshop discussions with presentations, which may have influenced participants' responses. Nonetheless, we feel that the PPI consultations have identified important insights that can now be further explored in future research.

Lastly, women with FGM are not a homogeneous group; FGM is a global issue, affecting women from different countries and cultures, with different needs according to their life stage. Therefore, to be fully representative, future PPIE work must engage a broader spectrum to ensure all voices are heard. Thus, consultations must be as accessible as possible, including with interpreters and childcare facilities, and events must be held at different times of day to suit women with young children and held across the United Kingdom.

## 6 Conclusions

Sexual expression, sexual pleasure and bodily autonomy are human rights. Reconstructive surgery can play a role in improving sexual function, body image and pain symptoms in women with FGM [19]. This PPIE work demonstrates that FGM survivors, as well as other stakeholders, feel this option should be explored, and transparency in service commissioning is required, given that similar services are currently available to other groups. Women with FGM have the right to holistic services, encompassing trauma counselling and psychosexual therapy, as well as suitable and safe surgical provision. This study highlights the need for robust further research in this area to help resolve this health inequity.

### 6.1 Extended Work and Future Research

ACERS is planning a co‐designed online survey based on what has emerged in this study, which will be translated into several languages and shared with multiple FGM community groups and social media platforms.

We are also planning to conduct a feasibility study.

## Author Contributions

**Juliet Albert:** conceptualisation, investigation, funding acquisition, methodology, validation, formal analysis, project administration, data curation, writing – original draft, writing – review and editing. **Janet Fyle:** writing – review and editing. **Njomeza Kartallozi:** writing – review and editing. **Christie Coho:** writing – original draft, writing – review and editing, formal analysis. **Naana Otoo‐Oyortey:** writing – review and editing. **Hekate Papadaki:** writing – review and editing. **Catrin Evans:** conceptualisation, writing – original draft, writing – review and editing, methodology, validation, formal analysis. **Dalia Saidan:** conceptualisation, writing – original draft, writing – review and editing, methodology, validation, formal analysis. **Sohier Elneil:** writing – review and editing. **Natasha Anderson‐Foster:** writing – review and editing, writing – original draft. **Jasmine Abdulcadir:** writing – review and editing. **Huda Mohamed:** writing – review and editing. **Aurora Almadori:** conceptualisation, investigation, writing – original draft, methodology, validation, writing – review and editing, formal analysis.

## Ethics Statement

The authors have nothing to report.

## Conflicts of Interest

The authors declare no conflicts of interest.

## Supporting information


**GRIPP2 LONG FORM**.

INVITATION TO FGM DISCUSSION WORKSHOPS.

EVENTBRITE INVITE TO 1^ST^ NATIONAL STAKEHOLDER EVENT.

**Post event survey ‐ Participant feedback after 2** ^**nd**^ **national stakeholder event**.

## Data Availability

The data that supports the findings of this study are available in the supporting material of this article.
