# Supplementary material for: Listening to Women's Voices: A Patient and Public Involvement Exercise Exploring Vulval Reconstructive Surgery for UK Women With Female Genital Mutilation (FGM)
Source: Health Expect. 2025 May 12;28(3):e70275. doi: 10.1111/hex.70275 (PMC12067389; doi:10.1111/hex.70275)
Supplement: Supplementary file 2 — INVITATION TO FGM DISCUSSION WORKSHOPS. [file HEX-28-e70275-s002.docx]

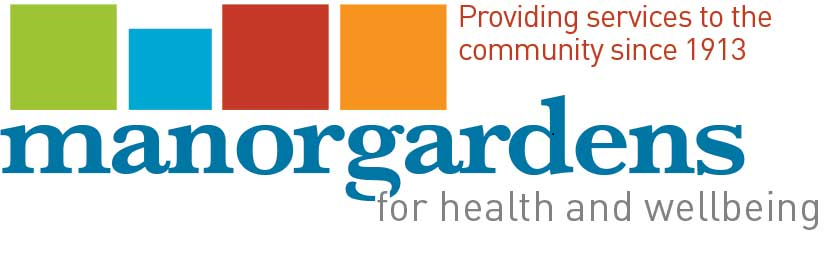


**You are invited to FGM Reconstruction discussion day**

**The event with take place at Manor Gardens Centre**

**10 Manor Gardens**

**Training Room**

**on**

**Wednesday**

**15^th^ May 2024**

**11 am – 2pm**

Refreshments will be provided

All women taking part will be given a gift of £50 One4all voucher

Travel costs up to £10 will be reimbursed with valid receipt

Please contact us if you need further details or to confirm attendance

Njomeza or Jenny

07483168774

07483334954

[njomeza@manorgardenscentre.org](mailto:njomeza@manorgardenscentre.org)

[jenny@manorgardenscentre.org](mailto:jenny@manorgardenscentre.org)

On arrival please press Training Room buzzer only
