# Supplementary figures and images for: Listening to Women's Voices: A Patient and Public Involvement Exercise Exploring Vulval Reconstructive Surgery for UK Women With Female Genital Mutilation (FGM)
Source: Health Expect. 2025 May 12;28(3):e70275. doi: 10.1111/hex.70275 (PMC12067389; doi:10.1111/hex.70275)

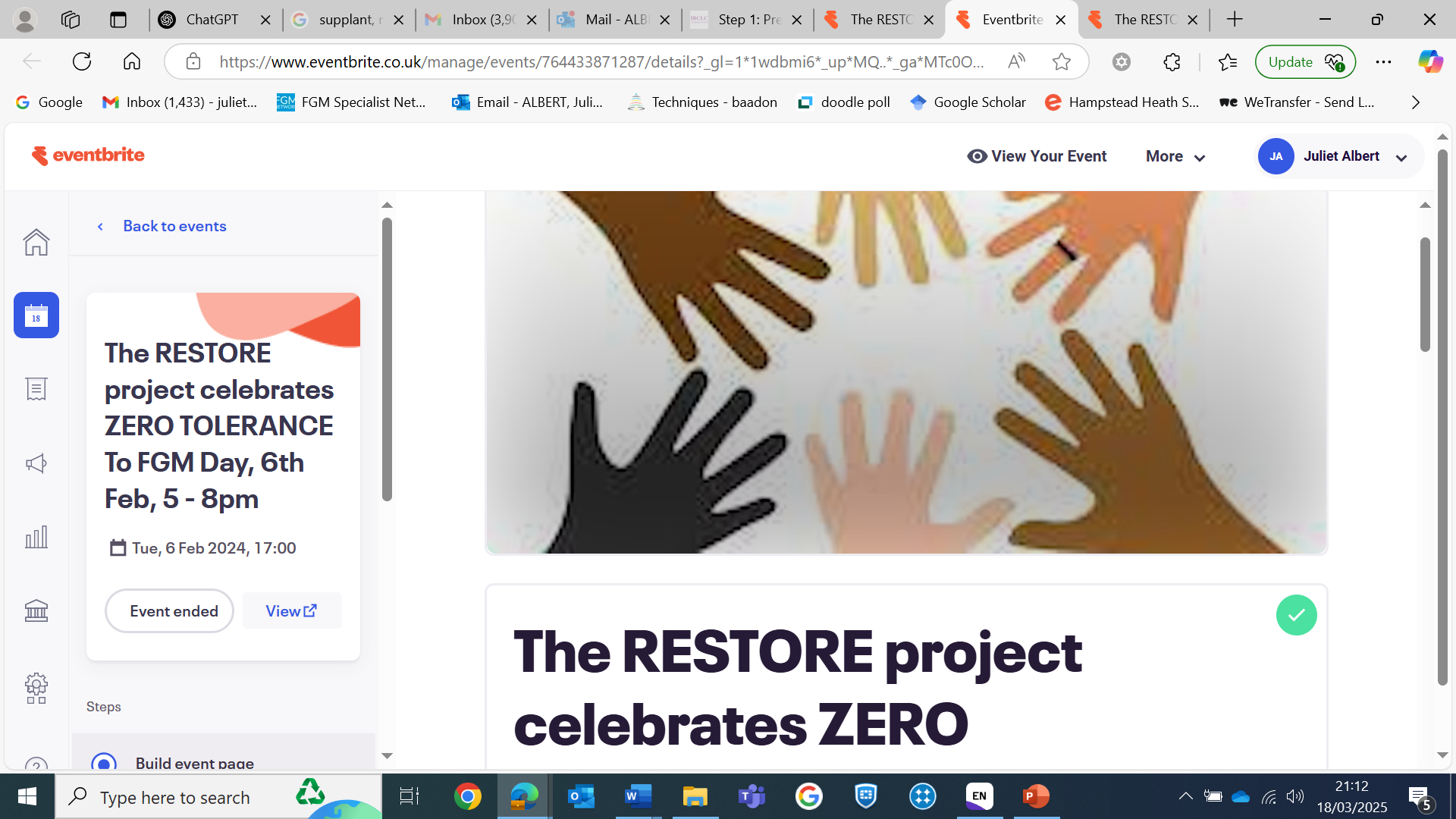

Supplement: Supplementary file 3 — EVENTBRITE INVITE TO 1ST NATIONAL STAKEHOLDER EVENT. [file HEX-28-e70275-s004.docx]
