# Supplementary material for: Listening to Women's Voices: A Patient and Public Involvement Exercise Exploring Vulval Reconstructive Surgery for UK Women With Female Genital Mutilation (FGM)
Source: Health Expect. 2025 May 12;28(3):e70275. doi: 10.1111/hex.70275 (PMC12067389; doi:10.1111/hex.70275)
Supplement: Supplementary file 4 — Post event survey ‐ Participant feedback after 2 nd national stakeholder event. [file HEX-28-e70275-s003.docx]

**Appendix 4. Post event survey - Participant feedback after 2^nd^ national stakeholder event**

|  | **Who are you? Or why did you**  **attend today?** | **What did you like most about the event?** | **What did you like least about the event?** | **How satisfied were you with the event? On a scale of 0-5 (5 is highest)** | **Do you have any suggestions for us to improve future events?** | **What city do you live in?** | **How old are you?** | **Any other comments or suggestions** | **How would you describe your ethnic background?** | **If you are an FGM survivor, would you be interested in having reconstruction surgery?** |
| --- | --- | --- | --- | --- | --- | --- | --- | --- | --- | --- |
| 1 | NGO/charity | Great speakers and so much informative information | Needed more time! But always the way in such interesting events - never enough time! | 5 |  | London | 32 | Amazing work. “Interpreters helps survivors feel included rather than always us speaking out for them. And I think it acknowledges that women here in the UK are sometimes still struggling with language.” | Afghan | No |
| 2 | trauma therapist/counsellor | Everything! | N/A | 5 |  | London | 31 |  | East African | No |
| 3 | allied health professional | Fantastic attendance and so many survivors present. Food was great | Brilliant event | 5 | No | London | 54 |  | White other | No |
| 4 | allied health professional | This event was inspirational and it was so amazing to be in a room | Nothing it was amazing | 5 |  | London | 31 |  | Mixed | No |
| 5 | Gynaecologist/  Obstetrician | Recon surgery, changing the narrative | No online access | 5 | Online option | West Sussex | 39 |  | African | No |
| 6 | FGM survivor | The atmosphere and what I learnt. | The time - would love a whole day conference! | 5 | Information on how to get involved - leaflets / posters  Opportunity to give feedback on the NHS service | London | 25 |  | Black - African - Sudanese | Maybe |
| 7 | FGM survivor | The honest truth about the procedure and knowing I can have the reconstruction | Nothing | 5 | Discussion should be included | London | 43 | Thank you all team | Somali | Maybe |
| 8 | FGM survivor | The information about organisation and the work dedicated to FGM and surgery solutions | The time | 5 | More time for the next event | London | 55 |  | Black African | No |
| 9 | trauma therapist/counsellor/FGM survivor | The diversity of participants, professional, lived experience, and professionals who have the service up and running in Geneva | Nothing , it was well organised. | 5 | Perhaps recording parts of the presentations? | Bristol | 36 | If there can be mobile events and gatherings in other cities to include women in other location. Or perhaps a possibility to join online | Arab | Maybe |
| 10 | specialist FGM Midwife | The presentation from Geneva - she spoke so well and it was very interesting. Also the food ;-) | Nothing | 5 |  | London | 38 |  | White British | No |
| 11 | allied health professional/FGM survivor | Hearing more about how the reconstruction surgery works and the progress ACERS has made | Nothing | 5 | Having it an option for virtual | London | 24 | Having Jasmine, a clinician who's been doing this for 10 years. She has data as well. It’s important to share this evidence - it’s not something that we’re just imagining | White British | Yes |
| 12 | Doctor | The positive vibe and the informal way to approach such a taboo topic. “It gives hope for the survivors who can see the other end, that change can happen”. | Nothing | 5 | Zoom/teams option, Music/dancing at the end (like the prev event), More visibility to the artist’s work (I.e. exposition closer to the refreshments) | London | 38 | Brilliant work! | White european | No |
| 13 | NGO/charity | Speakers, question and answers, refreshments | The chairs were unbelievably uncomfortable. | 5 | N/A | Birmingham | 50 | Lovely people and such a great learning and networking event | Black British Carribbean | No |
| 14 | Fgm safeguarding lead | Everything was amazing but I thought the survivors messages where powerful | N/a | 5 | None | Birmingham | 51 | Excellent event thank you | Black British Caribbean | No |
| 15 | NGO/charity/FGM survivor | I liked the whole evening. It was very informative and provided a great networking and discussions opportunity. I learnt a lot from the different presentations shared. And the food and venue were great too. | Maybe we needed more time to go into the discussions section. | 5 | Maybe we could start a bit early so we can have enough time for people to really get involved in the discussions and not rush to finish | London | 59 | Great event. We would like to organise a community event to have this information shared with community members. We have already discussed this with Juliet Albert. | Black African | Maybe |
| 16 | Doctor/FGM survivor | Reconstruction procedure | The time | 5 | If it will be Friday evening will be better | London | 36 | Thanks so much for your effort and support I had gynaecological problem need your help and advice. | Black African | Yes |
| 17 | FGM survivor | It was most informative. I was an hour late but found it exceptionally insightful. I was so encouraged by the friendliness and conversations afterwards. Everyone was there to help. | There was nothing to dislike. | 5 | No. | London | 51 | The women were all very kind. So were the men too. Thank you. And the lady at the reception desk was very welcoming too. Thank you to everyone. | South African - mixed | Yes |
| 18 | campaigner/activist | The atmosphere, the talks and the food | . | 5 | Perhaps have a day event, we have had it in the evening twice | London | 48 | For question 11, there should be a box to tick, N/A Rice, fish and salad needs to be on the menu at the next event | Black African | No |
| 19 | trauma therapist/counsellor | Presentations were informative with clear objectives. | I didn't have anything I didn't like | 5 |  | St Albans | 65 |  | South East Asian | No |
| 20 | from an FGM affected community | The event felt very soulful, and thoughtful. The music, art and food really set the scene and created a community atmosphere which felt very important when speaking about a whole person approach | Timing, the end felt quite rushed, it would have been nice to have split into smaller groups for more discussion | 5 |  | London | 33 |  | White British | No |
| 21 | trauma therapist/counsellor | How different organisations that work with FGM survivors came together. | Not keeping to time | 4 | Stick to timing | London | 56 | Thank you. | Black African | No |
| 22 | specialist FGM Midwife | Service User Experiences. So valuable to be able to hear their voices. As a senior manager it really does strengthen my ability to improve services but difficult as many communities are seldom heard. | All looked useful | 5 |  | Wakefield West Yorkshire | 52 | I was not able to attend the event (had my tickets booked but due to escalations at work I was not able to travel). However, I still found the slide decks really useful and informative (thank you for sharing). Really strong arguments regarding health inequalities especially compared to other forms of reconstructive surgery that is funded. Would definitely find another event useful. | White British | N/A |
| 23 | Midwife | Everything was very informative and useful | nothing | 5 | just follow up results | London | over 50 | wonderful event | Black Caribbean | No |
| 24 | health advocate/community champion; | great information ,great event | no every thing was | 5 | create more events in markets ,councils ,information stalls | london | 47 | thank you every thing was great | Black Arab | No |
| 25 | Professor of Urogynaecology and Uro-neurology; | The atmosphere and the enthusiasm of everyone participating! A great learning experience too | Nothing | 5 | Can we meet more frequently - perhaps 3 times regularly a year | London | Old enough! | Great event - would like more please | Black-Arab | No |
| 26 | From an FGM affected community | Varied professionals and their experience, opinions and expertise. | Nothing | 5 |  | London | 27 |  |  | No |
| 27 | Doctor; | The whole event was good, good to see new leadership from affected communities, excellent speaker from Geneva. | Nothing was bad. | 5 | Start on time and get the translation organised ahead of time. | nearest Southampton | 74 | occasional e-mails ( eg not more than monthly) only please and not from other organisations! | white british | No |
| 28 | From an FGM affected community;Gynaecologist/Obstetrician; | The diversity of the attendees. Moving forward in our thinking of restoration. | Opportunity to hear the voices of affected women. | 5 |  | West sussex | 39 |  | African | No |
| 29 | health advocate/community champion; campaigner/activist; work for an NGO/charity; | the approach beyond medical reasons | n/a | 5 | n/a | Oxford | 45 |  | white other | No |
